# Macrophage Plasticity in Reproduction and Environmental Influences on Their Function

**DOI:** 10.3389/fimmu.2020.607328

**Published:** 2021-01-14

**Authors:** Megan Chambers, April Rees, James G. Cronin, Manju Nair, Nicholas Jones, Catherine A. Thornton

**Affiliations:** ^1^Institute of Life Science, Swansea University Medical School, Swansea, United Kingdom; ^2^Maternity and Child Health, Singleton Hospital, Swansea Bay University Health Board, Swansea, United Kingdom

**Keywords:** macrophage plasticity, uterus, placenta, breast milk, infection, obesity, air pollution, immunometabolism

## Abstract

Macrophages are key components of the innate immune system and exhibit extensive plasticity and heterogeneity. They play a significant role in the non-pregnant cycling uterus and throughout gestation they contribute to various processes underpinning reproductive success including implantation, placentation and parturition. Macrophages are also present in breast milk and impart immunomodulatory benefits to the infant. For a healthy pregnancy, the maternal immune system must adapt to prevent fetal rejection and support development of the semi-allogenic fetus without compromising host defense. These functions are dependent on macrophage polarization which is governed by the local tissue microenvironmental milieu. Disruption of this microenvironment, possibly by environmental factors of infectious and non-infectious origin, can affect macrophage phenotype and function and is linked to adverse obstetric outcomes, e.g. spontaneous miscarriage and preterm birth. Determining environmental influences on cellular and molecular mechanisms that control macrophage polarization at the maternal-fetal interface and the role of this in pregnancy complications could support approaches to alleviating adverse pregnancy outcomes.

## Introduction

The establishment and ongoing success of pregnancy is reliant on finely tuned, dynamic maternal immune adaptations. It is critical that a balance between maintaining tolerance to the semi-allogenic fetus and upholding immune function for protection against infection is established to ensure a healthy pregnancy (1). A unique immunological crosstalk is established between mother and fetus which continues postpartum through breastfeeding. Important to this process of immune adaptation is the increase of innate immune cells at the maternal-fetal interface, specifically natural killer (NK) cells and macrophages, evident from the very beginning of pregnancy (2, 3). These macrophages and NK cells not only regulate local immune function but also directly promote migration and other functions of extravillous trophoblasts (EVT), support spiral artery remodeling and angiogenesis, and provide mediators that support fetal growth and development; all critical processes in placental and fetal development (4–6). Macrophages remain an important immune cell type to the maternal-fetal dyad post-partum as they exist in high numbers in breast milk and provide an element of protection against infection and inflammation to the nursing infant (7, 8).

Macrophages are found in all tissues where they play a role in maintaining tissue homeostasis and responding to the presence of infectious and non-infectious threat via the detection, ingestion and elimination of dead cells, foreign matter and other debris (9). They are a heterogenous population of immune cells displaying remarkable plasticity with their phenotype and function very much dependent on the local tissue microenvironment (6). Macrophages are classically divided into two groups; M1 macrophages (stimulated by interferon (IFNγ) or Toll-like receptor (TLR) ligands, such as lipopolysaccharide (LPS)) typically associated with pro-inflammatory responses and M2 macrophages (stimulated by interleukin (IL)-4/IL-13) typically associated with anti-inflammatory responses (10). While this has emerged as an over-simplification of the spectrum of macrophage phenotypes linked to specific effector functions it provides a starting point for discussing the role of macrophages in pregnancy. As such, the balance of macrophage polarization at the maternal-fetal interface has emerged as vital in sustaining a healthy pregnancy. Throughout gestation the number and proportion of M1/M2 macrophages at the maternal-fetal interface is finely tuned with the initial polarization state skewed towards M1 during the window of implantation contributing to the inflammatory response that is important for successful implantation (11). As pregnancy progresses M2-like macrophages are more abundant in order to establish and maintain tolerance to the fetus as well as contribute to the normal development and functioning of the placenta (5, 12, 13). Parturition has been characterized as an inflammatory process with macrophages being key to this process as demonstrated by increased numbers of M1 macrophages in the decidua of laboring tissue (14, 15). Failure to support the necessary immunological changes by macrophage maladaptation to or perturbations in the local tissue microenvironment, possibly due to alterations in the wider environment that the mother is being exposed to, is increasingly implicated in various pregnancy complications such as preeclampsia and preterm birth (1, 16).

This review will discuss the current understanding of the role of macrophages in the reproductive setting, namely the maternal-fetal interface as well as in breast milk, and the environmental factors that can influence their function including infection, obesity, and pollution.

## Macrophage Polarization and Plasticity

**Figure 1:**
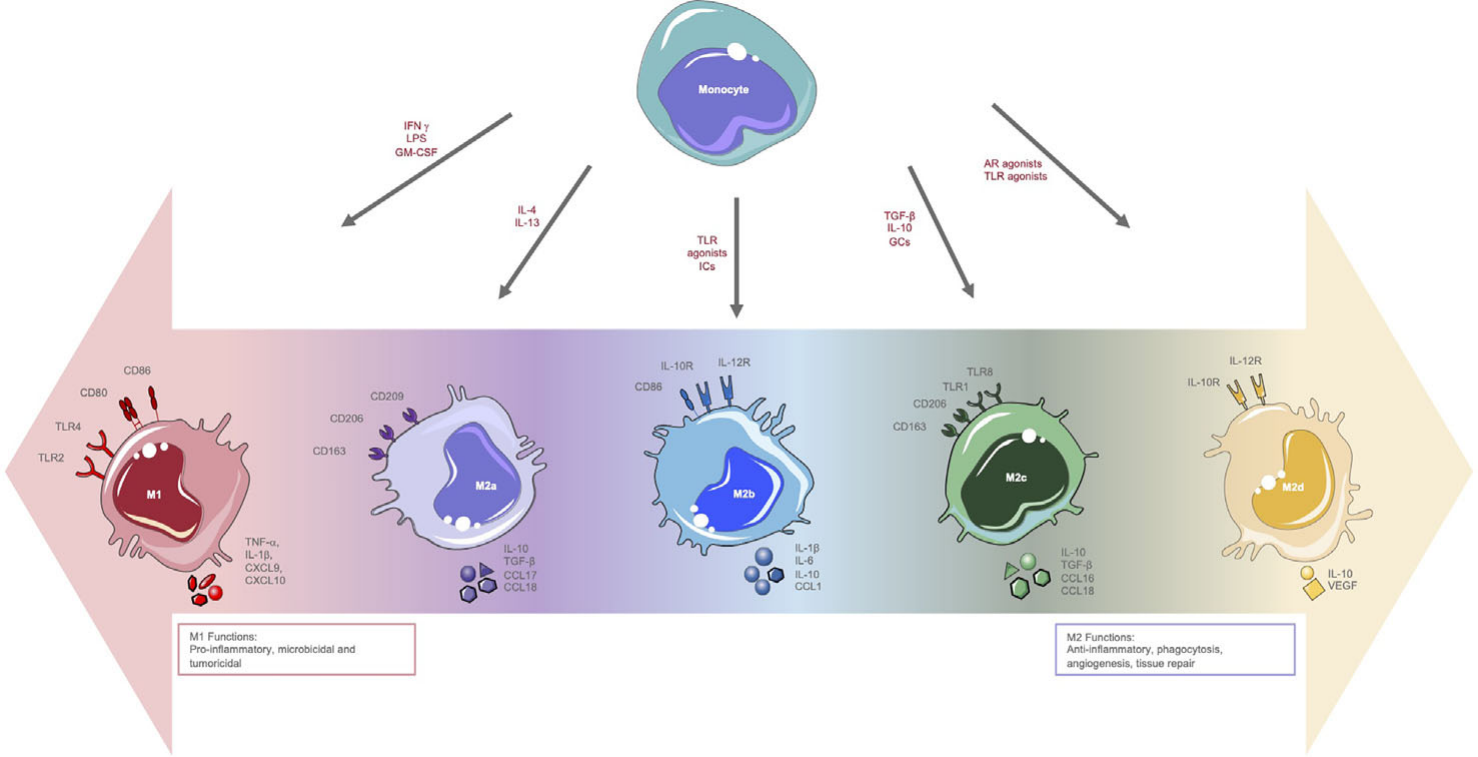
Macrophage polarization. Overview of the polarization spectrum of macrophages highlighting stimuli, surface marker expression, cytokine and chemokine secretion and functions of M1 and M2 polarized macrophages. AR, adenosine receptor; CCL, chemokine (C-C motif) ligand; CXCL, chemokine (C-X-C) ligand; IFNγ, interferon-gamma; GC, glucocorticoids; IC, immune complexes; LPS, lipopolysaccharide; MHC, major histocompatibility complex; TGFb, transforming growth factor-beta; TNFα, tumour necrosis factor alpha; TLR, Toll-like receptor; iNOS, inducible nitric oxide synthase; VEGF, vascular endothelial growth factor.

The diverse roles of macrophages are governed by their incredible plasticity. In response to extrinsic factors derived from the tissue microenvironment, macrophages activate different intracellular pathways leading to specific polarization patterns. As noted above, macrophages are categorized broadly into classically activated/M1 macrophages or alternatively activated/M2 macrophages and based on their response to differing stimuli M1 macrophages were suggested to preferentially elicit a Th1 type response and M2 a Th2 response (17–19). However, we now know that these classifications represent macrophages at either ends of a continuum and macrophage phenotype may be better described as a series of gradations within a large spectrum (20). Consequently, M2 macrophages have been further categorized into M2a, M2b, M2c, and M2d based on the molecules that lead to their activation as well as their gene expression profiles (21). The various groups of macrophages differ in their expression of cell surface markers, cytokine secretion and biological function which are summarized in **Figure 1**.

The activation of macrophages by LPS and Th1 cytokines (including IFNγ and TNFα) results in M1 polarization with granulocyte-macrophage colony-stimulating factor (GM-CSF) also implicated as an important M1-inducer (22). These macrophages are characterized by surface marker expression that includes CD80, CD86, TLR4, and TLR2 and the release of cytokines and chemokines such as TNFα, IL-1β, CXCL9, and CXCL10. These soluble mediators drive further M1 polarization via positive feedback, yielding potent pro-inflammatory cells with critical microbicidal and tumoricidal functions (**Figure 1**) (22). M2 polarization on the other hand is induced by signals from anti-inflammatory cytokines such as IL-4 and IL-13 as well as macrophage colony-stimulating factor (M-CSF) (22). M2 macrophages express surface markers such as CD206, CD209, and CD163 and upregulate the production of cytokines and chemokines such as IL-10, transforming growth factor (TGF-β), CCL1, and vascular endothelial growth factor (VEGF; **Figure 1**). M2 macrophages play important roles in tissue repair, angiogenesis, and immunomodulation (22).

A growing body of evidence demonstrates specific metabolic processes as critical determinants of immune cell effector functions including macrophages. A multitude of metabolic processes have been implicated in pro- and anti-inflammatory macrophage activation. These processes include glycolysis, the Krebs cycle, oxidative phosphorylation (OXPHOS), amino acid metabolism and fatty acid metabolism (23). Under homeostatic conditions and in an anti-inflammatory environment, macrophages rely primarily on catabolic pathways such as glucose oxidation for their energy supply and function (24, 25). Upon activation via pattern recognition receptor (PRR; e.g. TLR) signals, macrophages engage in anabolic metabolism in order to maintain inflammatory function (24, 26). Thus in M1 macrophages, aerobic glycolysis is recognized as a crucial metabolic event with inhibition of glycolysis affecting pro-inflammatory functions such as phagocytosis and cytokine release (27, 28). A high proportion of glycolysis-derived carbon is shunted into the pentose phosphate pathway to produce biosynthetic precursors required for nucleotide synthesis (pentose sugars and ribose-5-phosphate), and nicotinamide adenine dinucleotide phosphate (NADPH) which is required for reductive biosynthesis reactions and as a substrate for NADPH oxidase (NOX2) to generate ROS as part of their anti-microbial response (29, 30).

The Krebs cycle has emerged as a central immunometabolic regulator of macrophages, at least in mice, with specific breakpoints in the cycle resulting in accumulation of the intermediates succinate and citrate during pro-inflammatory macrophage activation. These metabolites are involved in the regulation of inflammatory gene expression (31, 32). The metabolite itaconate, which is synthesized from the Krebs cycle intermediate cis-aconitate, is also an important immunomodulator in M1 activated macrophages. Itaconate is produced in large quantities in LPS activated macrophages where it regulates inflammation by inhibiting pro-inflammatory cytokine and ROS production (33). Itaconate additionally displays antimicrobial properties by inhibiting bacterial isocitrate lyase (34). Another Krebs cycle intermediate, α-ketoglutarate, has been demonstrated as crucial for complete murine M2 macrophage activation via epigenetic alterations in M2-associated genes. These epigenetic alterations involve the demethylation of histone H3 K27 on the promoters of M2 marker genes thus enhancing their expression (35). However, while the Krebs cycle is implicated in both M1 and M2 polarization, it is intact in M2 cells with no breakpoints observed. M2 macrophage metabolism is also characterized by enhanced glucose oxidation via OXPHOS, with the anti-inflammatory cytokine IL-10 reported to stimulate OXPHOS to oppose M1 polarization (36–38). Originally, fatty acid oxidation (FAO) was thought to be important in supporting OXPHOS in these anti-inflammatory macrophages, however, the importance of FAO in M2 polarization is under scrutiny as many studies based this conclusion on inhibition of carnitine palmitoyltransferase-1A (facilitates long-chain fatty acid transport across the mitochondrial outer membrane) using etomoxir. However, high concentrations of etomoxir (exceeding 3 µM) have been demonstrated to disrupt free coenzyme A levels disrupting M2 polarization (39). The effect of FAO on murine macrophage polarization also has been studied using fatty acid transporter protein (FATP1) knockouts and overexpression models. FATP1 deficiency in macrophages induced a switch from FAO to glycolysis whereas overexpression inhibited pro-inflammatory macrophage responses supporting the hypothesis that FAO is important for anti-inflammatory M2 polarization (40). However, despite this evidence linking murine M2 polarization to FAO, FAO was found to be dispensable for M2 polarization of human macrophages and the role of FAO in human M2 polarization awaits clarification (41). Differential metabolism of the amino acid arginine might also contribute to M1/M2 polarization. M1 macrophages favor the inducible nitric oxide synthase (iNOS) pathway in which arginine is converted to citrulline and nitric oxide (NO) enhancing cytotoxicity, whereas M2 macrophages utilize the arginase pathway which involves the hydrolysis of arginine into urea and ornithine which is important for cell proliferation and tissue repair (42). While there are many fascinating insights into the link between macrophage metabolism and function the vast majority of macrophage metabolism studies have been carried out using murine cells and availability of human data is limited. Thus, more effort to understand the effects of metabolism on macrophage polarization in humans is needed.

The polarization patterns of macrophages within the female reproductive system varies throughout the menstrual cycle and during pregnancy. These patterns are dependent on gestational age, must be finely tuned to ensure pregnancy success and are perturbed in adverse pregnancy outcomes. Here we will provide an overview of human macrophage populations within the non-pregnant uterus (uterine macrophages), pregnant uterus (decidual macrophages), placenta (placental macrophages or Hofbauer cells) and breast milk. While placental macrophages are of fetal origin all other populations are of maternal origin.

### Uterine Macrophages

**Figure 2:**
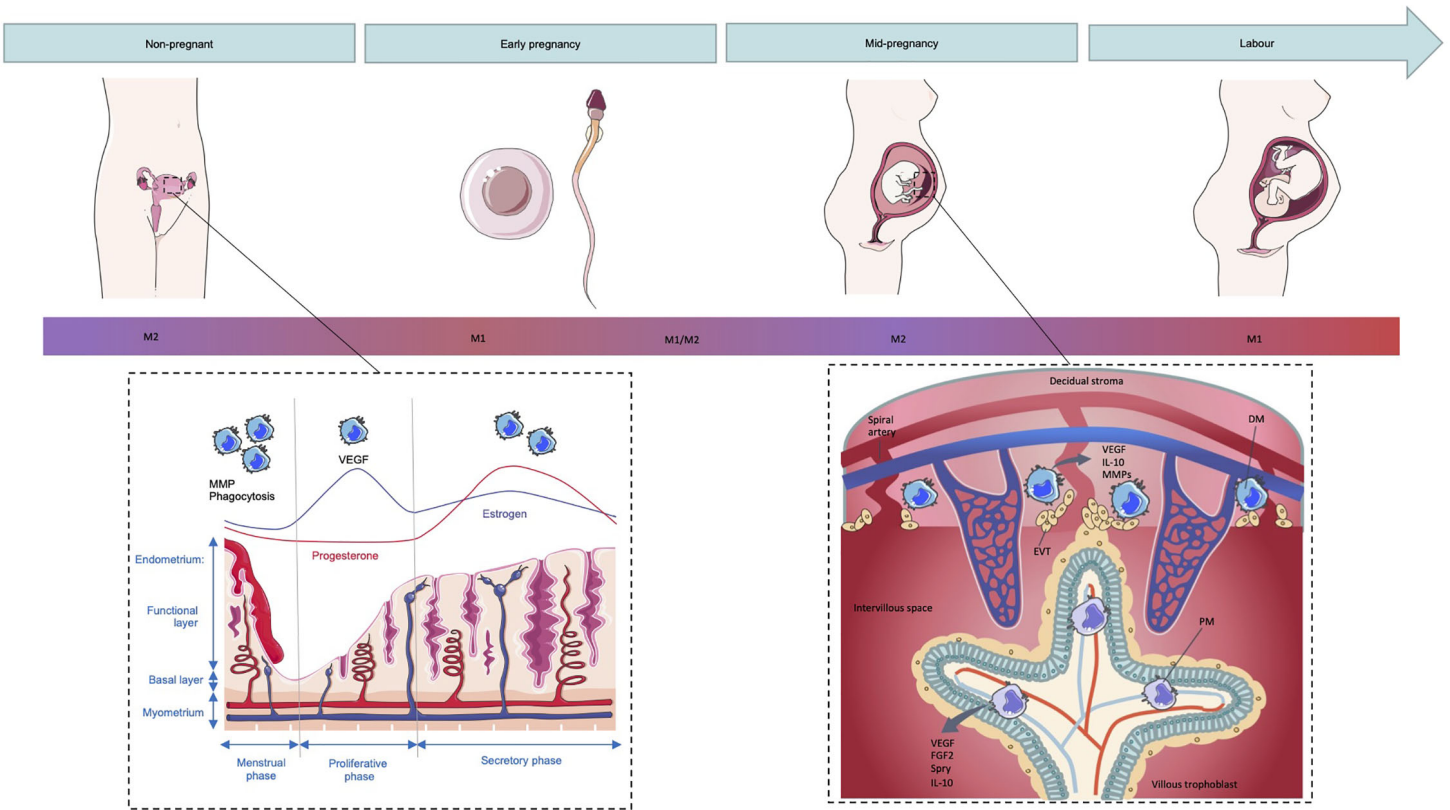
Macrophage polarization in the non-pregnant and pregnant uterus. The number and function of macrophages in the non-pregnant uterus changes during the menstrual cycle with numbers peaking during menses where they play a role in the breakdown of the endometrial functional layer through secretion of MMPs as well as debris clearance through phagocytosis. Upon exposure to seminal fluid, the endometrium is thought to induce an inflammatory response resulting in the recruitment of macrophages that display an M1-like phenotype. As pregnancy proceeds and extravillous trophoblasts (EVTs) invade the decidual stroma, decidual macrophages (DMs) shift towards a mixed M1/M2 profile and eventually a predominantly M2 phenotype to prevent fetal rejection. Placental macrophages (PMs) additionally display an M2 phenotype and aid in regulation of angiogenesis in the feto-placental vasculature through secretion of VEGF and fibroblast growth factor (FGF)2. Term labor is associated with an increased number of M1 macrophages and inflammatory cytokines in the decidua and it is this inflammatory environment which is thought to induce labor.

The uterine endometrium is an important site of mucosal immunity and must simultaneously maintain a hospitable environment for implantation while providing protection against pathogens. The physiological processes of the female reproductive system such as the menstrual cycle, implantation and the onset of labor all have been described as inflammatory events (43). Key to these inflammatory events are macrophages which are distributed throughout the uterine tissue and constitute about 10% of the leukocytes present in the human uterus and 10% of total uterine cells in the mouse (44, 45). These uterine macrophages have been demonstrated to display an M2-like phenotype characterized by high secretion of anti-inflammatory IL-10 and the typical M2 cell membrane markers CD163 and CD206 (46). This population of macrophages is likely regulated by the sex hormones estrogen and progesterone as inferred by the alterations in leukocyte populations which correspond to the menstrual cycle (**Figure 2**) (47, 48). Early murine studies revealed that ovariectomy, which prevents cyclical estrogen and progesterone production, leads to decreased macrophage numbers in the uterus within 6 days. Upon injection with estrogen and progesterone, macrophage numbers were restored highlighting a role for these hormones in macrophage regulation in the uterus (49). The macrophages in the uterine endometrium express the estrogen related receptor-β indicating the possibility of estrogen-dependent regulation of these cells (50). However, uterine macrophages lack progesterone receptor expression implying indirect regulation via factors secreted by other progesterone-responsive endometrial cells (51). Progesterone and estrogen additionally stimulate uterine cells to produce M-CSF and levels of this cytokine correlate to the presence of macrophages in the mouse uterus highlighting the role for sex steroids in controlling uterine macrophage numbers indirectly (52). The estrogen dominant follicular or proliferative phase of the menstrual cycle involves the proliferation and thickening of the endometrium. During the secretory or luteal phase of the menstrual cycle, in which progesterone dominates, decidualization involving morphological and functional changes of the endometrial stromal cells as well as spiral arteriole development to prepare for implantation begins (53). If implantation occurs, the decidualization reaction continues. However, in the absence of pregnancy, progesterone levels decrease resulting in an inflammatory cascade and the breakdown and loss of the functional layer of the endometrium in menses.

Despite the apparent M2 phenotype of uterine macrophages they secrete both anti-inflammatory (e.g., IL-10 and IL-1 receptor antagonist (IL-1RA)) and pro-inflammatory (e.g. TNFα and IL-1β) cytokines and express variations of surface markers throughout the menstrual cycle (54). Macrophage numbers are low during the proliferative phase and they express the activation markers CD69 and CD71 and the adhesion marker CD54 suggesting their potential involvement in the proliferation and regeneration of the functional endometrium (55). An influx of macrophages occurs in the secretory phase of the menstrual cycle where they are thought to be important in the preparation of the endometrium for implantation. Leukemia inhibitory factor (LIF) is a key molecule involved in implantation contributing through, for example, chemoattraction of macrophages. This role of LIF is best demonstrated in mouse models wherein LIF-knockout mice have a more than 50% reduction in macrophages in uteri that experience implantation failure (56).

The uterine macrophage population peaks during the menses phase following progesterone withdrawal (57). The decrease in progesterone levels as well as the release of paracrine factors from various cells of the endometrium results in the upregulation of matrix metalloproteinases (MMPs). These proteolytic proteinases are key to the breakdown of the endometrium during menstruation (58). Macrophages express various forms of these MMPs and additionally secrete various molecules involved in their regulation such as IL-17 which has been shown to upregulate MMP expression (59–61). Immunolocalization techniques have revealed that MMP-9 colocalizes with various leukocytes including macrophages immediately prior to and during menstruation suggesting that these immune cells are the major MMP-9 source in the menstrual tissue (61). MMP-12 is also secreted by uterine macrophages and is upregulated during menstruation highlighting the role of macrophage-derived MMPs in the tissue degradation associated with menstruation (62, 63). The endometrium resembles a “wound” during menstruation and it is critical that the wound-healing process maintains reproductive function (64). Inflammation is key to wound healing with macrophages having a pivotal role through removal of dead cells (64) and the remodeling of tissue (65). Endometrial cells shed during menstruation undergo apoptosis and are phagocytosed by macrophages (66). Subsequent vascular remodeling following progesterone withdrawal results in vasoconstriction and a hypoxic environment that creates proangiogenic macrophages and stimulates the release of VEGF by macrophages and stromal cells (67, 68).

Macrophages are therefore important for the normal functioning of the uterus throughout the menstrual cycle. Consequently, dysregulation of their activity seems to have a significant role in abnormalities and pathologies of the uterus including endometriosis and endometrial cancer (66). Endometriosis is a common condition affecting many menstruating women. It is characterized by hormone-dependent growth of vascularized endometrial tissue outside of the uterus resulting in pelvic pain and infertility. Macrophages are abundant in endometrial lesions found outside of the uterus (primarily in the peritoneum) and are implicated in endometriosis (69, 70). Macrophages within the peritoneum demonstrate increased secretion of pro-inflammatory cytokines (IL-1β, IL-6, and TNFα) and angiogenic factors (VEGF) contributing to a microenvironment that favors the implantation of endometrial cells outside of the uterus to establish and maintain endometriosis (71). The growth of endometrial lesions is likely enhanced by the presence of macrophages as demonstrated by the increased proliferation and invasiveness of endometrial stromal cells when co-cultured with macrophages (72). As well as endometrial lesion-associated macrophages in the peritoneum, macrophages were also increased in the endometrium of women with endometriosis during the proliferative phase of the menstrual cycle however alterations in their functions are yet to be identified (66). Elevated macrophage numbers in the endometrium of women with endometriosis may be due to the increased levels of macrophage migration inhibitory factor (MIF) and monocyte/macrophage activating chemoattractant protein (MCP-1/CCL2) that simultaneously limit macrophage migration from and recruitment of macrophages into the endometrium, respectively (73, 74). It is not clear whether these alterations in macrophages and other immune cell types implicated in endometriosis are cause or effect (75) but clarification of mechanisms mediating macrophage maladaptation could be critical in the development of treatment for endometriosis.

### Decidual Macrophages

As mentioned above, fluctuation of macrophage number and function in the endometrium is a physiological feature of the normal menstrual cycle in non-pregnant women (76). When pregnancy occurs there is an increase in the number of macrophages so that they comprise 20% to 30% of decidual leukocytes (77). During the peri-implantation period in mice, when the uterus is exposed to seminal fluid, there is an increase in M1-skewed macrophages (11) recruited by chemokines secreted by decidual stromal cells (78). The extent to which seminal fluid impacts the human endometrial environment is not well understood, however a similar inflammatory response is observed in the human cervix upon exposure to seminal fluid which results in the recruitment of leukocytes including macrophages, accompanied by inflammatory cytokines and chemokines such as IL-6 and IL-8 (CXCL8) (79). These immune changes are thought to facilitate preparation of the female reproductive tissue for pregnancy through clearance of debris and pathogens and sperm selection (80). Despite the primary site of semen deposition in the human female reproductive tract being the cervix, *in vitro* studies demonstrate that these immune changes probably extend to the uterus as seminal plasma was able to induce expression of proinflammatory cytokines in primary endometrial epithelial cells from fertile woman (81).

Although this initial inflammatory, pro-M1 period is key in the preparation for pregnancy, a shift towards a more immune tolerant environment must occur for pregnancy to continue (6). As EVTs begin to invade the uterine stroma, a mixed profile of M1/M2 macrophages is established (11). A shift towards a predominantly M2 phenotype then occurs in order to prevent fetal rejection (**Figure 2**) (6). However, the diverse phenotypes of decidual macrophages in the first and second trimester differ from conventional M1/M2 cells. Early murine studies demonstrated that macrophages, identified by expression of Fcγ receptor expression, isolated from the pregnant uterus were immunosuppressive as they were able to inhibit the inflammatory response of spleen cells to polyclonal mitogen phytohemagglutinin (PHA) suggesting that these macrophages may contribute to the immunoregulatory environment at the maternal-fetal interface (82).

Two distinct subsets of first trimester decidual macrophages classified by the level of expression of CD11c have been described (83). Gene expression analysis using RNA microarray revealed that CD11c^hi^ macrophages express genes involved in inflammation and lipid metabolism such as *OLR1* (oxidized low-density lipoprotein) and *LPL* (lipoprotein lipase). This subset was additionally much better at antigen processing and may therefore be the major antigen presenting population in the decidua. On the other hand, CD11c^lo^ macrophages upregulate expression of genes associated with extracellular matrix formation, muscle regulation, and tissue growth such as *DMD* (dystrophin), an important gene in muscle cell viability, and *IGF1* (insulin-like growth factor 1), a gene important for the development and functional maturation of skeletal tissues and reproductive organs (83). However, both of these decidual macrophage subsets secrete pro- and anti-inflammatory cytokines and might contribute to the inflammatory balance during the first trimester that maintains immune homeostasis without compromising defense against invading pathogens at the maternal-fetal interface (83). In contrast, gene expression profiling and phenotyping by surface marker expression of term decidual macrophages demonstrates that they most closely resemble M2 skewed cells (12, 15, 84). This phenotype is important for immunomodulatory functions and tissue remodeling (12). These term decidual macrophages are the major source of the immunosuppressive cytokine IL-10 in the decidua and display low expression of CD80 and CD86 (co-stimulatory molecules necessary for antigen presentation and T-cell activation) thus confirming their immunomodulatory role (85). As well as IL-10, there are many other factors at the maternal-fetal interface that are responsible for modulating this phenotype. For example, trophoblast-derived M-CSF along with IL-10 have been demonstrated to induce this M2 regulatory phenotype in maternal monocytes (86). Soluble human leukocyte antigen G5 (sHLAG5), a soluble isoform of human leukocyte antigen, has additionally been implicated as an important soluble factor responsible for macrophage polarization during pregnancy. sHLAG5 is able to promote differentiation of macrophages to an immunomodulatory phenotype with reduced expression of CD86 and increased CD163 expression (87). These macrophages additionally displayed increased phagocytic ability as well as greater expression of indoleamine 2,3-dioxygenase [a marker of decidual macrophages (85)] and secretion of IL-6 which prevented proliferation of and IFN-γ production by T cells.

Decidual macrophages have been reported to be involved in various stages of placental development. They contribute to decidual invasion of EVT from placental villi and uterine spiral artery remodeling that support the nutritional and oxygen demands of the growing fetus. As with endometrial remodeling in the menstrual cycle the production of MMPs, specifically MMP-7 and -9, is critical as these degrade the extracellular matrix enhancing EVT invasion (88). Additionally, the secretion of M2-associated factors such as IL-33 and Wnt-5a by decidual macrophages may enhance proximal cell column proliferation and could be important for EVT development (89). Macrophages also localize in the immediate vicinity of spiral arteries even before EVTs are present highlighting their role in early vascular remodeling (88).

Apoptosis is central to appropriate development of the decidua (90), for example, trophoblast cells lost via apoptosis are replaced with a younger population (91), and macrophages engulf these apoptotic cells preventing the release of their potentially pro-inflammatory and pro-immunogenic intracellular contents that might be lethal for the fetus (90). Thus, appropriate removal of apoptotic cells by macrophages and potentially the production of anti-inflammatory mediators such IL-10 and TGF-β in response to apoptotic cells (92) is important for maintaining tissue homeostasis at the maternal-fetal interface.

While an M2-like decidual macrophage phenotype dominates for much of pregnancy, labor at term is associated with an increased number of M1 macrophages when compared with term in the absence of labor suggesting a role for pro-inflammatory macrophages in the induction of term labor (15). Additionally, parturition is associated not only with the infiltration of macrophages but also increased mRNA expression of the pro-inflammatory cytokines *IL1B* and *IL6* in laboring tissue (93). However, an earlier study identified only marginal differences in macrophage activation status between spontaneous vaginal delivery compared with caesarean section questioning the involvement of decidual macrophage activation in parturition (84).

### Placental Macrophages

Placental macrophages, also known as Hofbauer cells, are fetal macrophages found within the chorionic villi. They appear as early as the 18th day of gestation and are round, highly vacuolated cells (94). As placental macrophages are observed before the fetal circulation is established, it is suggested that they are derived from mesenchymal cells in the villous stroma from early stages of gestation (95–97). A recent study additionally concluded that first trimester placental macrophages were derived from primitive hematopoiesis as they are transcriptionally similar to yolk sac derived macrophages but not embryonic monocytes (98). Other studies have suggested that upon development of the fetal circulation, placental macrophages are recruited from fetal monocytes in later stages of pregnancy as transitional forms of monocytes and placental macrophages are observed (99–101). The pool of placental macrophages is thought to be sustained through recruitment of monocytes by factors such as MCP-1 produced by villous fibroblasts (102). It is additionally debated whether placental macrophages are able to self-renew with some studies demonstrating mitotic activity (103) while others did not (104). Placental macrophages also have been shown to display an M2-like phenotype. M1-associated genes such as *TLR9*, *IL1B, IL12RB2*, and *CD48* were silenced by hypermethylation whereas pro-M2 genes including *CCL2, CCL13, CCL14*, and *CD209* were hypomethylated (105). Flow cytometry confirms the presence of the M2 cell surface markers CD209 and CD206 on placental macrophages and supernatants from these cells have high concentrations of immunosuppressive cytokines such as IL-10 and TGF-β (106, 107). This M2 phenotype aids in the prevention of fetal rejection and allows for fetal growth (107) and is dictated by the local microenvironment. For example, M1 polarized monocyte-derived macrophages favor the M2 phenotype upon co-culture with placental mesenchymal stem cells isolated from the chorionic villi (108).

The phenotype and function of placental macrophages remains poorly understood and has recently been debated due to the finding that a population of maternal derived macrophages is present in placental digests that have likely been unaccounted for in previous studies (98). Despite this, a number of studies support a key role for them in angiogenesis, vasculogenesis, and placental mesenchyme development (94, 109, 110) which remains true even with the removal of this contaminating population of maternal macrophages (98). The placenta is a highly vascularized organ and placental macrophage secretion of VEGF and fibroblast growth factors (FGFs) such as FGF2 underscores their likely role in the regulation of angiogenesis in the feto-placental vasculature (109, 111). Additionally, placental macrophages express Sprouty (Spry) proteins which are involved in regulating branching morphogenesis and attracting fibroblasts to support this process (102). Although characterized as M2-like, placental macrophages can mount a pro-inflammatory response upon activation via TLRs (98, 112). TLRs are a group of transmembrane proteins that function as PRRs, recognizing pattern-associated molecular patterns (PAMPs) and danger-associated molecular patterns (DAMPs) (113). Activation of term placental macrophages via TLR4 (with LPS) or TLR3 (with polyinosinic-polycytidylic acid (poly I:C)) increased IL-6 and IL-8 secretion suggesting that placental macrophages have an important role in host defense within the placenta and the triggering of local inflammation (112). Placental macrophage anti-viral responses have been studied further using 5′ triphosphate double-stranded RNA (5′ppp-dsRNA) which is a synthetic ligand for the retinoic acid-inducible protein I (RIG-I) (114), another type of PRR. There are gestational differences in the capacity of placental macrophages to respond to 5′ppp-dsRNA. Early/mid gestation placental macrophages quickly adopted classically activated phenotypes with the production of some inflammatory cytokines whereas term macrophages remained outwardly inactivated despite the transcriptional upregulation of the antiviral genes *MX1* and *Viperin* (114). These results suggest that placental macrophage response to viral stimulation may be temporally regulated across gestation. First trimester placental macrophages additionally demonstrate microbicidal activity as they display phagocytic capacity and ROS production (98).

### Breast Milk Macrophages

Macrophages continue to have an important role even after the baby is born via the breast milk. Breast milk is composed of many immunological factors which facilitate immune development of the neonate (115). Macrophages are present in breast milk in large numbers comprising up to 80% of total cells in early human milk with numbers per milliliter of milk declining with milk maturation (116). These cells are thought to be derived from maternal peripheral blood monocytes which extravasate and migrate to the mammary gland and enter the breast milk through the epithelium. Upon arrival, breast milk macrophages demonstrate great phagocytic activity without prompting a significant, uncontrolled immune reaction that would result in inflammation and tissue damage (117).

Although monocytes are thought to be the precursors of breast milk macrophages, these two populations have distinct phenotypes. Breast milk macrophages are larger, contain numerous cytoplasmic inclusions and display increased expression of molecules involved in T cell stimulation, including HLA-DR, CD86, and CD40 (116). The glycoprotein CD83, a molecule expressed by mature dendritic cells (DCs) and in activated cells of other lineages such as neutrophils (118), is expressed by breast milk macrophages but not monocytes (116). These results suggest that breast milk macrophages are committed to DC differentiation. This is also supported by their spontaneous production of GM-CSF, the most potent monocyte-derived DC differentiation factor. Indeed, upon exposure to IL-4 breast milk macrophages can differentiate into DCs able to stimulate T cells and potentially mediate T cell dependent immune responses (116). Breast milk macrophages also secrete the multifunctional protein osteopontin which activates Th1 cells highlighting the role for these macrophages in T cell modulation (119). A Th1 gastrointestinal environment in the infant has been implicated in the prevention of allergies however clear evidence is limited due to environmental influences on individual levels of any mediators measured in breast milk (119). Breast milk macrophages can survive in the neonatal murine gut for several hours with some mucosal uptake suggesting a direct contribution of breast milk macrophages to gastrointestinal immune response in the offspring (120). However, the function and transfer of breast milk macrophages in human infants remains poorly understood.

Breast milk macrophages are also suggested to have a host defense role. An increase in breast milk macrophage numbers beyond what is considered normal at the different lactation stages occurs with infection in both the mother and infant (121, 122). However, as well as this protective role, breast milk macrophages might contribute to mother to child transmission of pathogens (123). For example, TLR10 is especially overexpressed on breast milk macrophages from women infected with HIV-1 compared with uninfected women. TLR10 contributes to HIV-1 infection and replication in breast milk macrophages through interaction with the HIV-1–specific structural protein gp41 leading to the production of IL-8 (124).

### Environmental Factors Implicated in Adverse Obstetric Outcomes

**Figure 3:**
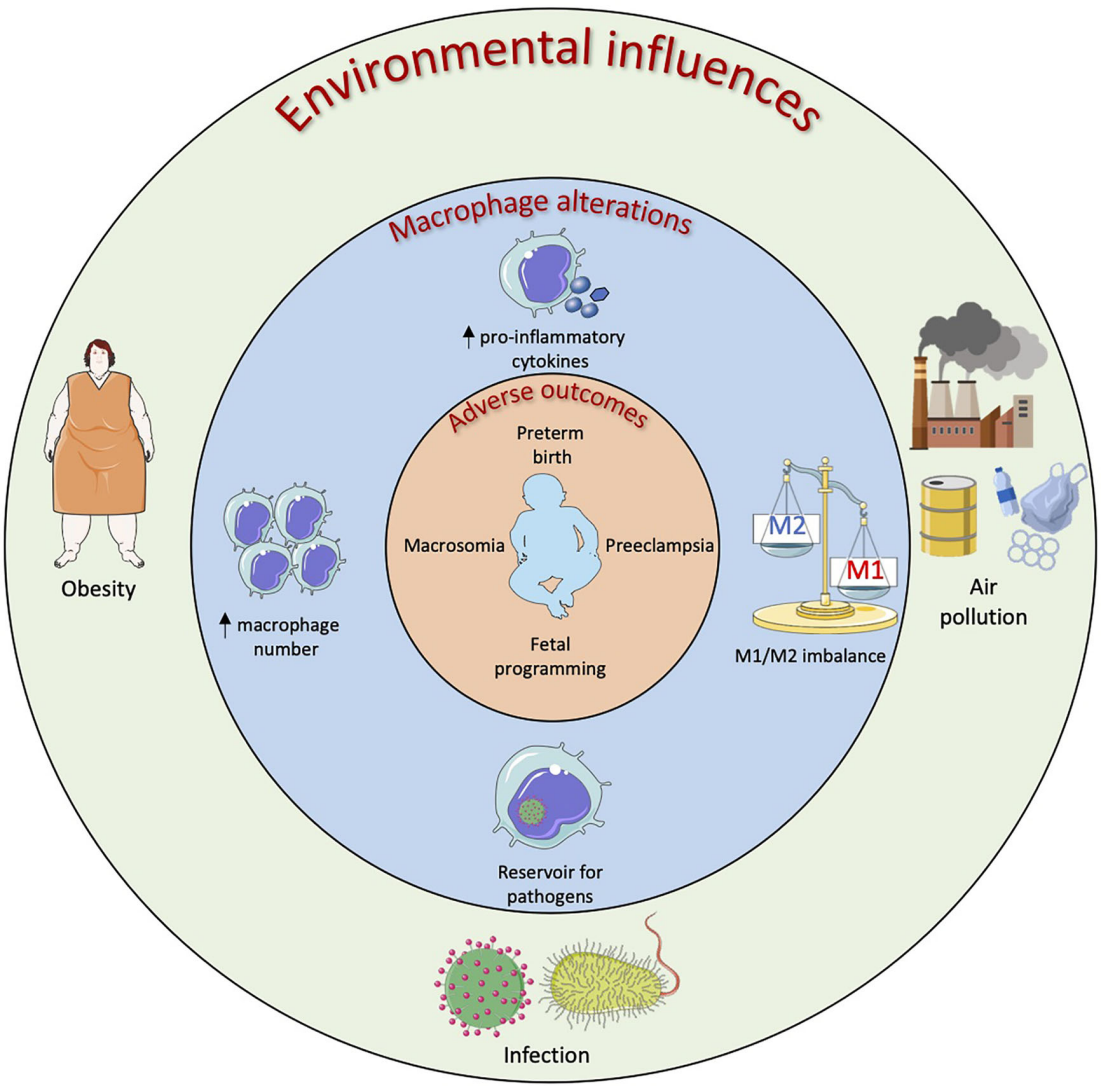
Environmental influences on macrophages in the female reproductive system resulting in adverse obstetric outcomes. Maternal obesity, infection and air pollution have all been linked to adverse obstetric outcomes and this could be linked to macrophage alterations at the maternal-fetal interface. Obese mothers have demonstrated increased numbers of placental macrophages along with increased levels of pro-inflammatory cytokines. This placental inflammation has been linked to preeclampsia, preterm birth and fetal programming of organ function. In utero infection by bacteria and viruses can result in neural defects, preterm and stillbirth. Placental and decidual macrophages have been shown to act as reservoirs for these pathogens as well as upregulate their pro-inflammatory cytokine production resulting in an imbalance of M1/M2 macrophages at the maternal-fetal interface. Air pollutants such as black carbon and chemicals found in plastics that are inhaled as well as ingested can cross the placental barrier and be phagocytosed by macrophages. The effects of air pollution on placental/decidual macrophage function are yet to be fully elucidated however certain pollutants have been shown to increase pro-inflammatory cytokine production as well as increase prostaglandin production which could lead to preterm birth.

The central role of macrophages is to respond to the local tissue environment to maintain and restore tissue homeostasis including mounting an inflammatory response to pathogenic and non-pathogenic insult. Consequently, macrophages are uniquely positioned to respond to maternal environmental pressures with infection and obesity among the most studied but with increasing interest in other factors such as air pollution. The effects of environmental influences on macrophage phenotype and function at the maternal-fetal interface and resulting adverse outcomes are summarized in **Figure 3**.

#### Infection

Infection is included here as the best studied environmental influence to provide a comparison to the other environments of interest rather than an extensive review. The immune response during pregnancy is finely controlled to allow close contact between maternal and fetal cells. However, maternal systemic infections as well as ascending infections from the vagina can result in placental dysfunction and thereby adverse pregnancy outcomes (**Figure 3**). Viruses and bacteria can reach the decidua and placenta through hematogenous transmission or by ascending from the lower reproductive tract (125). Infection by the family of TORCH organisms which include Toxoplasma, Others (syphilis, varicella-zoster, parvovirus B19), Rubella, Cytomegalovirus (CMV), and Herpes as well as Hepatitis E virus (HEV-1) and Zika virus (ZIKV) during pregnancy can result in severe maternal and/or fetal morbidity (126). Decidual and placental macrophages have been proposed as a line of defense against these pathogens however progressive research has demonstrated that these macrophages rather harbor live pathogens serving as a reservoir for fetal infection (127, 128). These invading pathogens manipulate the macrophages resulting in perturbed function. For example, infection of placental macrophages by ZIKV results in cell proliferation and hyperplasia of these cells (129). ZIKV infection also increased secretion of pro-inflammatory cytokines, such as IFNα and IL-6, and chemokines such as MCP-1, implicated in monocyte infiltration, and IP-10, involved in effector T cell recruitment (128). The putative migratory abilities of these infected placental macrophages might facilitate the spread of the virus to the fetal brain resulting in neural abnormalities and microcephaly (130).

Aberrant macrophage activation also occurs with *Toxoplasma gondii* infection during pregnancy, especially if this occurs during the first trimester, and can lead to neural defects as well as stillbirth, miscarriage and preterm labor (131). A number of studies have demonstrated aberrant decidual macrophage activation upon infection with *T. gondii*. Expression of M1-associated molecules, such as CD80 and CD86, was upregulated and M2 functional molecule expression was down-regulated in infected decidua (131, 132). *T. gondii* infection also increased pro-inflammatory cytokine secretion from decidual macrophages which have been shown to induce trophoblast apoptosis (131, 133). Altered balance of M1 versus M2 decidual macrophages might contribute to *T. gondii*-mediated adverse obstetric outcomes by dysregulating immune tolerance.

Placental macrophages can however provide some protection against certain infections. For example, they have been found to limit HIV-1 replication and possibly contribute to reduced mother to child transmission despite the expression of HIV-1 co-receptors such as CD4 and DC-SIGN on their surface (107). This limited replication is thought to be due to the increased production of immunoregulatory cytokines such as IL-10 by these placental macrophages as IL-10 has been shown to inhibit HIV-1 replication (107).

Therefore, macrophages at the maternal-fetal interface are a double-edged sword during infection as polarization towards an M1 microbicidal phenotype will result in compromised tolerance and threat to fetal health however an anti-inflammatory M2 phenotype compromises maternal and fetal protection from pathogens.

#### Obesity

Obesity in women of child-bearing age is increasing worldwide. The 2019 National Maternity and Perinatal Audit Clinical Report reports that more than half (50.4%) of UK women were overweight or obese at the time of initial antenatal booking appointment and an estimated obesity prevalence of 31.8% in women aged 20–39 years was recorded in the United States in 2012 (134, 135). Obesity has been associated with infertility as well as a number of obstetric complications such as spontaneous miscarriage, preeclampsia and macrosomia (**Figure 3**) (134). The mechanisms underpinning this are not completely understood but as obesity demonstrates characteristics of a low-grade inflammatory state (136), an altered immune balance likely contributes. Little data is available on the role uterine macrophages play in infertility in obese woman however a study on women with polycystic ovary syndrome (which is associated with infertility) revealed that obesity induces an inflammatory environment with increased numbers of macrophages and TNFα signaling in the endometrium of these women (137). This inflammatory environment could contribute to fertility failures in these women. Additionally, obesity is a risk factor for the gynecological disorder known as adenomyosis which is characterized by invasion of endometrial glands and stroma within the uterine myometrium (138, 139). Women with adenomyosis have reduced IVF implantation rates and it has been demonstrated that there is an increased density of macrophages and NK cells in these women suggesting successful implantation in these women may be hindered through an immunological mechanism (140). Placental macrophages are emerging as having a role in this with increased numbers in placentas from obese compared with normal weight women and an accompanying augmentation of the pro-inflammatory cytokines TNFα, IL-1, and IL-6 (141). However, not all studies report an increase in placental macrophages with maternal obesity although greater expression of pro-inflammatory cytokines in placentas of obese women is emerging as a consistent finding (142). Placental macrophages therefore seem to be contributing to the inflammatory environment observed in chronic villitis associated with maternal obesity (143). Placental macrophages express PRRs such as TLR4 and can bind saturated fatty acids to induce production of potent pro-inflammatory mediators such as TNFα, IL-6, and IL-8 (144, 145). An *in vitro* model of obesity using high levels of glucose, insulin and palmitic acid (saturated lipid) in culture additionally demonstrated that palmitate alone was enough to cause NLRP3 inflammasome activation, resulting in the release of IL-1β, as well as induce placental macrophage apoptosis (146). This placental inflammation might have negative impacts on the development of the fetus, including the brain, resulting in long term alteration in neural function (147).

The effects of obesity on decidual macrophage populations have also been studied. Macrophage populations in the decidua parietalis of obese women show fewer M1 macrophages but no change in M2 macrophages compared with healthy controls (148). This decrease in M1 macrophages in the decidua parietalis might be a compensatory mechanism for the heightened inflammatory state observed in obesity. While only term placentas from uncomplicated pregnancies were included in this study, it was suggested that failure of this compensatory mechanism could lead to adverse pregnancy outcomes associated with obesity (148).

Obesity has been considered a risk factor for preeclampsia, a condition characterized by new onset hypertension and proteinuria (149). Preeclampsia is associated with deficient spiral artery remodeling and is linked to altered numbers of placental immune and trophoblast cells (149). Many mechanisms linking the two conditions have been proposed including that insulin resistance and hyperinsulinemia (commonly observed in obesity) precede the clinical manifestation of preeclampsia (150). Additionally, increased insulin levels in pregnant rodents led to raised blood pressure (151). Metabolic alterations associated with obesity including hyperinsulinemia, hyperleptinemia and hyperlipidemia all affect placental function and perfusion (152). Increased levels of low-density lipoproteins (LDL) and triglycerides have been observed in women with preeclampsia and LDLs have been reported to reduce trophoblast migration (153). Increased numbers of decidual macrophages from patients with preeclampsia that were localized to the spiral arteries whereby trophoblast invasion was reduced also has been demonstrated (154). While apparently paradoxical, this increase in placental macrophages might accompany the increased apoptosis of placental trophoblasts implicated in preeclampsia pathogenesis (155). This likely reflects increased sensitivity of EVTs to Fas-mediated apoptosis (156) with decidual macrophages in preeclamptic placentas displaying increased Fas-ligand expression potentiating their ability to induce Fas-mediated apoptosis of the Fas-expressing trophoblasts and limiting trophoblast invasion (155). Restricted trophoblast invasion leads to decreased spiral artery remodeling and reduced uteroplacental blood flow. TNFα has a key role in this increased apoptosis of trophoblast implicating M1-like macrophages (157). Local enhanced levels of GM-CSF, a potent inducer of M1 macrophages (see **Figure 1**), occurs in the preeclamptic placenta and might underpin changes in macrophage phenotype and function (158). However, the increase in pro-inflammatory cytokines will stimulate decidual cells to upregulate factors, such as M-CSF, that induce M2 polarization for increased phagocytosis of the apoptotic trophoblasts (159).

Studies on the effects of maternal obesity on breast milk macrophage phenotype and function are limited. Two recent *ex vivo* studies revealed that breast milk macrophages from mothers with high BMI displayed reduced phagocytic ability as well as reduced ROS production upon exposure to zymosan particles (160, 161). These results suggest that breast milk macrophages from obese mothers might be less efficient at responding to infection.

#### Air Pollution

Tobacco smoke exposure is the best studied maternal inhaled exposure and the detrimental effects of tobacco smoking on pregnancy outcomes such as miscarriage, stillbirth and preterm birth are well recognized (162). Many molecules in tobacco cigarettes can cross the placental barrier and affect placental function and fetal development but the specific causal mechanisms are not completely understood (163). Term placental macrophages become dysfunctional upon *in vitro* exposure to cigarette smoke extract (164). Phagocytosis is compromised and increased release of TNFα and IL-33 but decreased IL-10 potentially perturbs the immune tolerance ecosystem within the placenta leading to adverse pregnancy outcomes. Increasing epidemiological and experimental evidence highlight an association between other air pollution exposures during fetal development and similar adverse obstetric outcomes including low birth weight, preterm birth and preeclampsia as well as long term-term complications for the offspring (165, 166). While numerous studies have described these associations, little is known about the mechanisms underpinning these adverse outcomes. Both indirect (such as respiratory and intrauterine inflammation) and direct (such as particle translocation) processes probably contribute. Black carbon, as found in particulate matter (PM), has been observed in the human placenta where it seems to localize to and be phagocytosed by placental macrophages (167, 168). PM negatively affects placental trophoblast cells with endocytosis-mediated accumulation of PM associated with increased IL-6 production and decreased cell growth linked to decreased human chorionic gonadotropin (hCGβ) (169). Other phenotypic responses of trophoblast exposed to PM related to molecular transport, cell survival and inflammation were reminiscent of those described in preeclampsia and intrauterine growth restriction but effects of PM on human placental macrophages are unknown. Mouse placental macrophage exposure to PM significantly increased gene expression of inflammatory markers such as *IL-1B* and oxidative stress markers such as heme oxygenase 1 (170) further highlight the need to study the effects of PM on human placental macrophages.

Studies on the effects of other pollutants on reproductive tract macrophages are emerging. Diethylhexyl phthalate (DEHP) is a pollutant that can be found in dust and can be inhaled. DEHP is commonly used in the production of polyvinyl chloride (PVC) products such as pipes, medical devices and automotive parts. If DEHP is not covalently bonded to the PVC it can be released into the environment. The active metabolite of DEHP is mono-2-ethylhexyl phthalate (MEHP) and exposure to this metabolite during the prenatal period is associated with increased risk of preterm birth (171). MEHP has been found in maternal serum, placental tissue, fetal cord serum and amniotic fluid indicating that the metabolites of DEHP are transferable to the fetus from maternal blood (172). Exposure of placental macrophages to MEHP resulted in significantly increased prostaglandin E2 (PGE_2_; a labor inducer) levels perhaps providing a link between DEHP exposure, placental macrophages and preterm birth (173).

Environmental pollutants can additionally be transferred from mother to infant postnatally through the breast milk (174). One example of these toxic compounds is the metal barium which is present in natural sources such as rocks and contaminate drinking water or can be released into the environment through industry (175). Barium chloride nanoparticles have toxic effects on breast milk phagocytes including reduced cell viability and intracellular calcium release which is important in promoting cell activity (175). Exposure to barium chloride also increased apoptosis in breast milk phagocytes suggesting that reduced numbers might reach the infant and therefore negatively affect their immune development.

While data on the effects of air and other pollutants, other than environmental tobacco smoke, on different reproduction-associated macrophages is nascent there is clearly a need to investigate these interactions given the long-term health detriments for the fetus of exposures during pregnancy.

## Discussion

Macrophages are significant contributors to immune function and homeostasis at the maternal-fetal interface. Compelling evidence suggests that these cells are involved in various stages of reproduction including implantation, placentation, pregnancy maintenance, parturition and in the development of the neonatal immune system through breastfeeding. The ability of macrophages to perform this plethora of functions is due to their high plasticity, with their phenotype governed by the local tissue microenvironmental milieu. Precise regulation of macrophage polarization is necessary for successful pregnancy. Alterations in macrophage polarization due to environmental influences are proposed in pregnancy complications however the mechanisms involved have not been elucidated fully.

Macrophage plasticity along with their significant role in directing pregnancy outcomes offers potential therapeutic targets in pregnancy complications. Modulating macrophage phenotypes through immunotherapy could prevent adverse pregnancy outcomes. The modulation of macrophage phenotype using epigenetic modifiers has been carried out with success in inflammatory disease models such as acute lung injury in mice (176). Epigenetic modulators could have therapeutic potential in ameliorating adverse obstetric outcomes, but one has to question what affect these epigenetic alterations might have on the baby. Recent data has provided insight into the contribution of cellular metabolic pathways such as glycolysis and oxidative phosphorylation to macrophage phenotype plasticity (23). Therefore, there is scope to therapeutically target these events too as already postulated for autoimmune diseases and cancer (177–179). For example, dimethyl fumarate (DMF), a cell permeable derivative of fumarate, targets and inactivates the glycolytic enzyme glyceraldehyde 3-phosphate dehydrogenase (GAPDH) downregulating glycolysis and preventing activation of macrophages leading to a balance of inflammatory and regulatory cells (178). DMF is used to treat relapsing multiple sclerosis by inhibiting pro-inflammatory pathways and offers potential for treating the pro-inflammatory profile of many adverse pregnancy outcomes. However, again application in the reproductive setting might be challenging and we lack fundamental information about the relative contribution of different cellular metabolic processes to macrophage function at different sites and at different stages of pregnancy as well as in breast milk postnatally. Effort needs to be made to elucidate the signaling pathways and specific mechanisms controlling macrophage polarization in reproductive tissues for fundamental insight and translation for the development of therapeutics to counter adverse obstetric and longer-term health outcomes.

A potentially less hazardous approach to modifying reproductive macrophage behavior might be through lifestyle modification and nutraceutical approaches to achieve local microenvironmental changes that translate as change to macrophage function. Perhaps therefore the most effective way to avoid adverse obstetric outcomes due to environmental influences would be to educate women as to the effects of these factors and highlight the importance of a healthy lifestyle to minimize obesity, exposure to infection and environmental pollutants prior to conception and during pregnancy and breastfeeding.

## Author Contributions

MC and CT conceived and designed the review. MC wrote the first draft of the manuscript. AR, NJ and CT wrote sections of the manuscript. MN provided clinical oversight. JG and NJ provided oversight of immunometabolism. All authors contributed to the article and approved the submitted version.

## Funding

MC is funded by Swansea University Research Excellence Scholarship. Work in the group is supported by Ser Cymru II, Welsh Government.

## Conflict of Interest

The authors declare that the research was conducted in the absence of any commercial or financial relationships that could be construed as a potential conflict of interest.
